# The application value of targeted next-generation sequencing in the diagnosis of primary osteoarticular infections: A single-center study

**DOI:** 10.3389/fcimb.2025.1593228

**Published:** 2025-09-18

**Authors:** Zhengyong Tao, Mengqi Zhu, Jiandang Shi, Zongqiang Yang, NingKui Niu

**Affiliations:** ^1^ Department of Orthopedic, General Hospital of Ningxia Medical University, Yinchuan, China; ^2^ Ningxia Medical University, Yinchuan, China; ^3^ Research Center for Prevention and Control of Bone and Joint Tuberculosis, General Hospital of Ningxia Medical University, Yinchuan, Ningxia, China

**Keywords:** targeted next-generation sequencing (TNGS), primary osteoarticularinfection (POI), diagnostic value, pathogen detection, drug resistance gene

## Abstract

**Objective:**

To evaluate the diagnostic performance and clinical utility of targeted next-generation sequencing (tNGS) in primary osteoarticular infections (POI).

**Methods:**

Eighty-seven patients diagnosed with POI at the Bone Infection Ward of Ningxia Medical University General Hospital between September 2023 and September 2024 were enrolled, including cases of tuberculous osteoarticular infection (35 cases), Brucella-related osteoarticular infection (21 cases), and pyogenic osteoarticular infection (31 cases). Using bacterial culture, Xpert MTB/RIF assay, Brucella agglutination test, and histopathological examination as reference standards, the diagnostic value of tNGS in pathogen identification and resistance gene analysis was systematically evaluated.

**Results:**

All patients had complete follow-up data. The cohort comprised 87 POI patients (mean age: 55.36 ± 17.24 years; male-to-female ratio: 1.35:1). tNGS demonstrated significantly higher overall sensitivity than conventional bacterial culture (85.0% *vs*. 31.0%, *P* < 0.001). For resistance profiling, tNGS identified *Mycobacterium tuberculosis* complex mutations associated with resistance to isoniazid (2 cases), rifampicin (2 cases), ethambutol (1 case), pyrazinamide (5 cases), and streptomycin (1 case). Additionally, one fluoroquinolone resistance gene and one extended-spectrum β-lactamase (ESBL)-producing pathogen were detected. Notably, one multidrug-resistant (MDR) case harbored mutations conferring resistance to five anti-tuberculosis agents. Receiver operating characteristic (ROC) curve analysis revealed that tNGS exhibited superior diagnostic accuracy for tuberculous osteoarticular infections (AUC = 0.926), Brucella-related osteoarticular infections (AUC = 0.891), and pyogenic osteoarticular infections (AUC = 0.912), outperforming Xpert MTB/RIF (0.814), Brucella agglutination test (0.832), bacterial culture (0.652), and histopathology (0.704) (all *P* < 0.05).

**Conclusion:**

tNGS enables simultaneous pathogen identification and resistance gene detection with high efficiency, broad coverage, and accuracy, demonstrating significant advantages in POI diagnosis. This technology holds critical value in guiding optimized antimicrobial therapy and is recommended as a first-line molecular diagnostic tool for POI.

## 1 Introduction

Osteoarticular infections, as a significant type of infectious diseases, pose severe challenges in clinical management. Primary osteoarticular infections (POI) specifically refer to the initial infections originating in the osteoarticular system, which can affect the limbs, joints, spine, and other areas. Epidemiological studies indicate that spinal infections, as a particular type of POI, have reached an annual incidence rate of 6.5 per 100,000 in Europe and the United States, demonstrating a continuous upward trend (Babic and Simpfendorfer, 2017). The pathogenic mechanism of this disease primarily involves hematogenous dissemination, however, in approximately 30% of cases, the source of infection remains unclear (Bagcchi, 2023). From an etiological perspective, bacterial infections predominate, accounting for 85%-90% of cases. Among these, purulent bacteria (such as *Staphylococcus aureus*, *Streptococcus* spp.) comprise approximately 65%, while granulomatous infections (such as *Mycobacterium tuberculosis*, *Brucella* spp.) account for 20%-25%. Although fungal infections represent less than 5% of cases, they often lead to misdiagnosis and catastrophic consequences due to the biological characteristics of pathogens like Aspergillus and Candida (Henry et al., 2017).

The clinical manifestations of POI (Primary Osteoarticular Infection) are predominantly nonspecific, with typical symptoms including persistent localized pain (92%), restricted mobility (78%), and elevated inflammatory markers. Notably, patients with immunocompromised states (such as diabetes, HIV infection, solid organ transplantation, etc.) have an increased risk of infection dissemination by 3-5-fold (Jeon and Murray, 2008). In terms of diagnostic strategies, current guidelines emphasize a multimodal evaluation system, which constructs a diagnostic framework by combining clinical presentation, imaging characteristics (with MRI having a sensitivity of 94%), and microbiological evidence. However, etiological confirmation still faces significant bottlenecks: traditional bacterial culture is limited by a long detection period (average 5–7 days) and difficulty in detecting anaerobic bacteria, with a false negative rate reaching 40%-60% in patients exposed to antibiotics; serological tests (such as the Brucella agglutination test) although easy to perform, carry a risk of false positives due to cross-reactivity (specificity only 82%-88%) (Dorman et al., 2018). Although molecular assays such as Xpert MTB/RIF significantly enhance the diagnostic efficiency for osteoarticular tuberculosis, they are unable to simultaneously detect non-tuberculous mycobacteria and drug resistance profiles.

Against this backdrop, next-generation sequencing technologies offer a novel approach to overcoming diagnostic challenges. Metagenomic next-generation sequencing (mNGS), through unbiased pathogen screening, has demonstrated a sensitivity of 83.6% in the identification of spinal infection pathogens, representing a 27.3% improvement over traditional culture methods (Deurenberg et al., 2017). However, currently, the economic cost of metagenomic next-generation sequencing is significantly higher than that of culture and histopathology, which limits its widespread application in clinical practice (Xu et al., 2022). In comparison, targeted next-generation sequencing (tNGS) offers advantages such as shorter turnaround time, relatively lower cost, and high sensitivity in etiological diagnosis. tNGS is a next-generation sequencing technology that has been developed by combining targeted enrichment techniques with high-throughput sequencing. Through tNGS, it is possible to detect dozens to hundreds of known pathogenic microorganisms and their virulence or drug resistance genes. Targeted next-generation sequencing (tNGS), employing a multiplex PCR amplification strategy, can specifically capture over 400 orthopedic-related pathogens and 23 categories of drug resistance genes, achieving a detection rate of 89.2% in the diagnosis of periprosthetic infections, with an average detection period reduced to 26 hours (Wilkes, 2023). Current evidence indicates that tNGS has established a mature clinical application pathway in the field of pulmonary infections, with a consistency rate of 93.7% with drug susceptibility testing (Deng et al., 2023; Gaston et al., 2022). tNGS can also reliably predict *Mycobacterium tuberculosis* drug resistance directly from clinical specimens or cultures and provide timely and critical information for the appropriate treatment of patients with tuberculosis (Murphy et al., 2023; Zhang et al., 2022). In periprosthetic infections, tNGS was superior to bacterial culture in detection of pathogenic bacteria, not statistically significantly different from mNGS, and superior to bacterial culture as well as mNGS in detection of resistance genes as well as cost and turnaround time (Huang et al., 2023). For POI, pathogenesis as well as the detection and diagnosis of resistance genes are equally important. However, high-quality research in the field of bone and joint infections remains scarce, particularly lacking a systematic evaluation of the pathogen spectrum characteristics specific to primary osteoarticular infections (POI).

This study, based on the aforementioned clinical needs, is the first to systematically evaluate the comprehensive efficacy of targeted next-generation sequencing (tNGS) in the pathogen diagnosis and drug resistance detection of primary osteoarticular infections (POI). By constructing a multi-pathogen control study model, it aims to provide high-level evidence for optimizing the diagnostic pathway for osteoarticular infections.

## 2 Materials and methods

### 2.1 Inclusion and exclusion criteria

Inclusion Criteria:

Patients diagnosed with primary osteoarticular infection based on clinical symptoms, signs, laboratory tests, and imaging examinations.
Adequate tissue samples obtained through surgery or ultrasound-guided puncture for tNGS testing.
Patients who underwent relevant etiological examinations: bacterial culture, X-pert, Brucella agglutination test, and pathological examination.
Exclusion Criteria:
Diagnosed patients who did not receive relevant treatment.
Follow-up duration of less than 1 month.
Patients with severe immune system diseases (such as advanced cancer, HIV/AIDS, etc.).
Patients with incomplete clinical data.

### 2.2 General clinical data

According to the inclusion and exclusion criteria, 87 cases of POI diagnosed in the bone infection ward of Ningxia Medical University General Hospital from September 2023 to September 2024 were included. The average age was 55.36 ± 17.24 years, with a male-to-female ratio of 1.35:1, including osteoarticular tuberculosis (35 cases), Brucella osteoarthritis (21 cases), and pyogenic osteoarticular infection (31 cases). Data on patient age, gender, underlying diseases, clinical symptoms, laboratory tests, and imaging results were collected. Tissue samples were obtained intraoperatively in patients undergoing surgery, and by percutaneous puncture in those managed non-surgically. Each sample was divided into three portions: the first portion was placed in a sterile transport container for pathogen culture and X-pert testing; the second portion was stored in a sterile transport container for tNGS testing; and the third portion was fixed with formalin for histopathological analysis. This study was approved by the hospital’s ethics committee, and all patients signed informed consent forms.

#### 2.2.1 Bacterial culture

Tissue specimens were ground into a homogenate and inoculated on blood agar, chocolate, and MacConkey plates for bacterial culture, and Sabouraud agar for fungal culture. Joint fluid specimens were directly injected into aerobic and anaerobic blood culture bottles for testing and culture.

### 2.3 Xpert (using nested real-time fluorescent quantitative PCR method)

Tissue samples were collected from the patient’s lesion through surgery or puncture. The samples were mixed with the buffer in the kit for preliminary processing. The processed samples were placed in the Xpert device, and amplification was carried out using PCR technology, with real-time monitoring of the reaction and analysis of the results.

### 2.4 Brucella agglutination test (SAT)

#### 2.4.1 Sample collection

Serum samples were collected from the individuals to be tested, usually venous blood.

#### 2.4.2 Serum dilution

The serum samples were mixed with diluent at a certain ratio (usually 1:10, 1:20, etc.) and incubated at room temperature for a certain period (such as 30 minutes).

#### 2.4.3 Antigen addition

Prepared Brucella antigen was added to the diluted serum and gently mixed.

#### 2.4.4 Incubation

The mixed liquid was incubated at an appropriate temperature (usually 37 °C) for a certain period (such as 2 hours or overnight) to observe agglutination.

#### 2.4.5 Result observation

The presence of agglutination at the bottom of the test tube was observed with the naked eye or under a microscope.

### 2.5 Pathological examination

#### 2.5.1 Sample collection

Tissue specimens were obtained through surgical resection, biopsy, etc.

#### 2.5.2 Fixation

The collected tissue samples were immediately fixed using 10% formalin solution to preserve cell morphology and structure and prevent decay.

#### 2.5.3 Dehydration and embedding

The fixed samples underwent dehydration using different concentrations of ethanol and were then embedded in paraffin for slicing.

#### 2.5.4 Slicing

The paraffin-embedded tissues were cut into thin slices using a microtome, usually 4–5 micrometers in thickness.

#### 2.5.5 Staining

The slices were stained to facilitate clear observation under a microscope. Common staining methods include HE staining (Hematoxylin-Eosin staining) and immunohistochemical staining.

#### 2.5.6 Microscopic examination

The stained slices were examined under a microscope to observe cell morphology, arrangement, and tissue structure for diagnosis.

### 2.6 Targeted next-generation sequencing

#### 2.6.1 Library construction and sequencing

The library fragment size and concentration were quality-controlled using the Agilent 2100 Bioanalyzer and Qubit dsDNA HS Assay Kit (Thermo Fisher Scientific Inc.). The GenSeizer targeted capture and library construction technology, developed by Shanghai Binyuan Medical, was used. Firstly, a large number of primers targeting the genes of interest were used to perform ultra-multiplex PCR amplification on the nucleic acids in the test samples to achieve targeted enrichment of the gene amplification products. Then, specific sequencing adapters and tags were added to the amplification products of different samples through a round of PCR to complete the library construction. The DNB library was denatured into single-stranded circular DNA through rolling circle replication amplification to generate DNB nanoballs. After loading onto the sequencing chip, sequencing was performed using the MiniSeqDx-CN sequencing platform. High-throughput sequencing platform (Guangzhou Jinyu Ruike Sequencing Platform).

#### 2.6.2 Bioinformatics analysis

**Figure 1:**
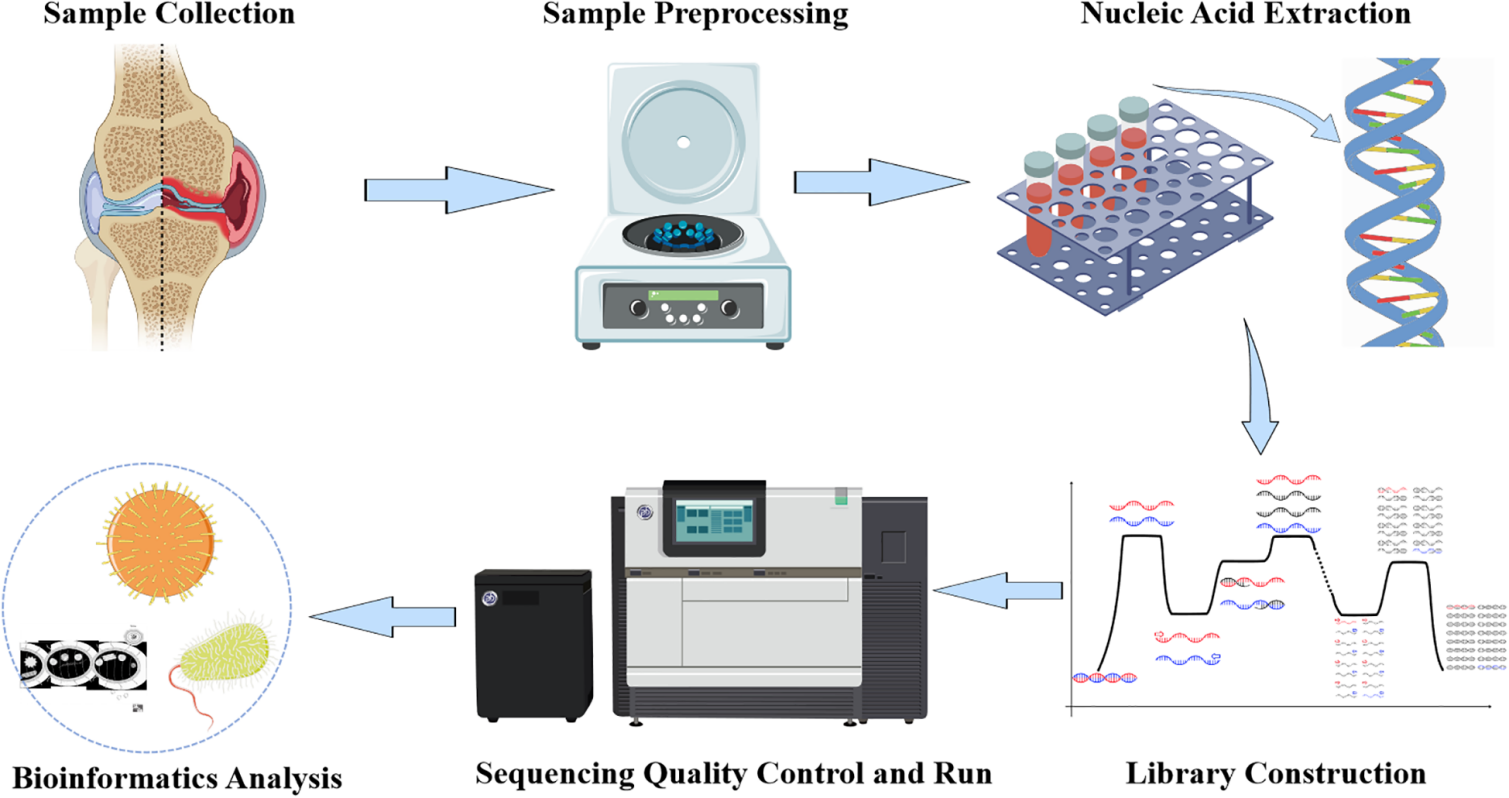
tNGS workflow diagram.

Low-quality and short read sequences (length <35bp) were removed from the sequencing data using Burrows-Wheeler Alignment (BWA: http://bio-bwa.sourceforge.net/). The remaining read sequences were compared with a pathogen-specific microbial database, which includes over 4000 clinically common pathogenic microorganisms, including bacteria, fungi, parasites, DNA viruses, RNA viruses, etc., as well as important bacterial resistance genes and virulence genes, to provide rapid identification. Read counts that could match various pathogen-specific sequences were obtained through comparative algorithms, and potential pathogens were determined based on sequence quantity and other clinical tests (**Figure 1**).

### 2.7 Statistical analysis

Data analysis was performed using SPSS version 26.0. Categorical variables are presented as percentages, and continuous variables are expressed as means with standard deviations. Sensitivity, specificity, positive predictive value (PPV), and negative predictive value (NPV) were calculated with 95% confidence intervals. Normality of continuous variables was assessed using the Shapiro-Wilk test. Inter-group comparisons were conducted using the Chi-square test, and the consistency of tNGS with other testing methods was evaluated by the Kappa test. The diagnostic performance of different methods was compared using the Delong test. A P-value of <0.05 was considered statistically significant.

## 3 Results

### 3.1 Characteristics of the study population

This study included 87 patients with primary osteoarticular infection (POI), with an average age of 55.36 ± 17.24 years, consisting of 50 males (57.5%) and 37 females (42.5%).

#### 3.1.1 Based on etiological classification

Tuberculous osteoarticular infection: 35 cases (40.2%), with an average age of 57.20 ± 18.31 years, including 17 males (48.6%) and 18 females (51.4%);

Brucellar osteoarticular infection: 21 cases (24.1%), with an average age of 57.67 ± 10.54 years, including 14 males (66.7%) and 7 females (33.3%);

Pyogenic osteoarticular infection: 31 cases (35.6%), with an average age of 51.71 ± 19.40 years, including 19 males (61.3%) and 12 females (38.7%) (**Table 1**).

**Table 1:** Inclusion criteria for patient characteristics.

Characteristic	Total	TB	B0i	PJI
Age(year)	55.36 ± 17.24	57.20 ± 18.31	57.67 ± 10.54	51.71 ± 19.40
Gender	87	35	21	31
Male	50(57.4%)	17(48.6%)	14(66.7%)	19(61.3%)
Female	37(42.6%)	18(51.4%)	7(33.3%)	12(38.7%)
DM	11(12.6%)	2(5.71%)	3(14.3%)	6(19.4%)
T-spot	87	35	21	31
Positive	45(51.7%)	30(85.7%)	8(38.1%)	7(22.6%)
Negative	42(48.3%)	5(14.3%)	13(61.9%)	24(77.4%)
SAT	87	35	21	31
Positive	20(23.0%)	0	19(90.1%)	1(3.2%)
Negative	67(77.0%)	35(100.0%)	2(9.5%)	30(96.8%)
Bacterial Culture	87	35	21	31
Positive	27(31.0%)	0	10(47.6%)	16(51.6%)
Negative	60(69.0%)	35(100.0%)	11(52.4%)	15(48.4%)
Pathology	87	35	22	31
Positive	24(27.6%)	22(62.9%)	2(9.1%)	0
Negative	63(72.4%)	13(37.1%)	20(90.9%)	31(100.0%)

### 3.2 Pathogen detection spectrum and test consistency

#### 3.2.1 Pathogen detection spectrum

**Figure 2:**
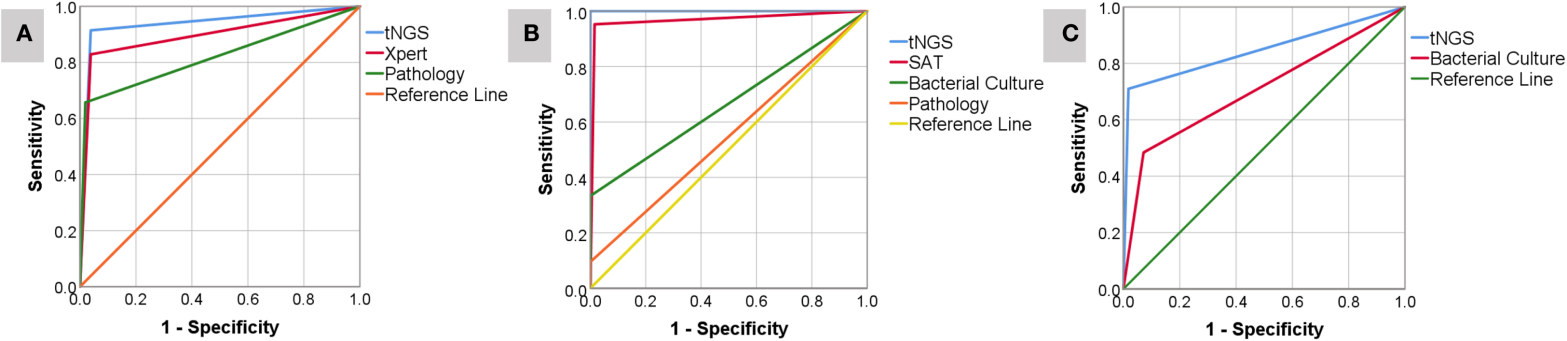
ROC curve analysis of the value of tNGS with POI diagnostic indicators **(A-C)**.

The microbial results detected by tNGS and culture in this study indicated that *Mycobacterium tuberculosis* complex was the most common microorganism detected by tNGS (33 cases), followed by Brucella (21 cases). Other detection results are shown in the figure. The number of pathogens detected by tNGS and culture showed that bacteria are the most common pathogens in primary osteoarticular infections, followed by viruses and fungi (**Figure 2**).

#### 3.2.2 Consistency of tNGS and culture

**Figure 3:**
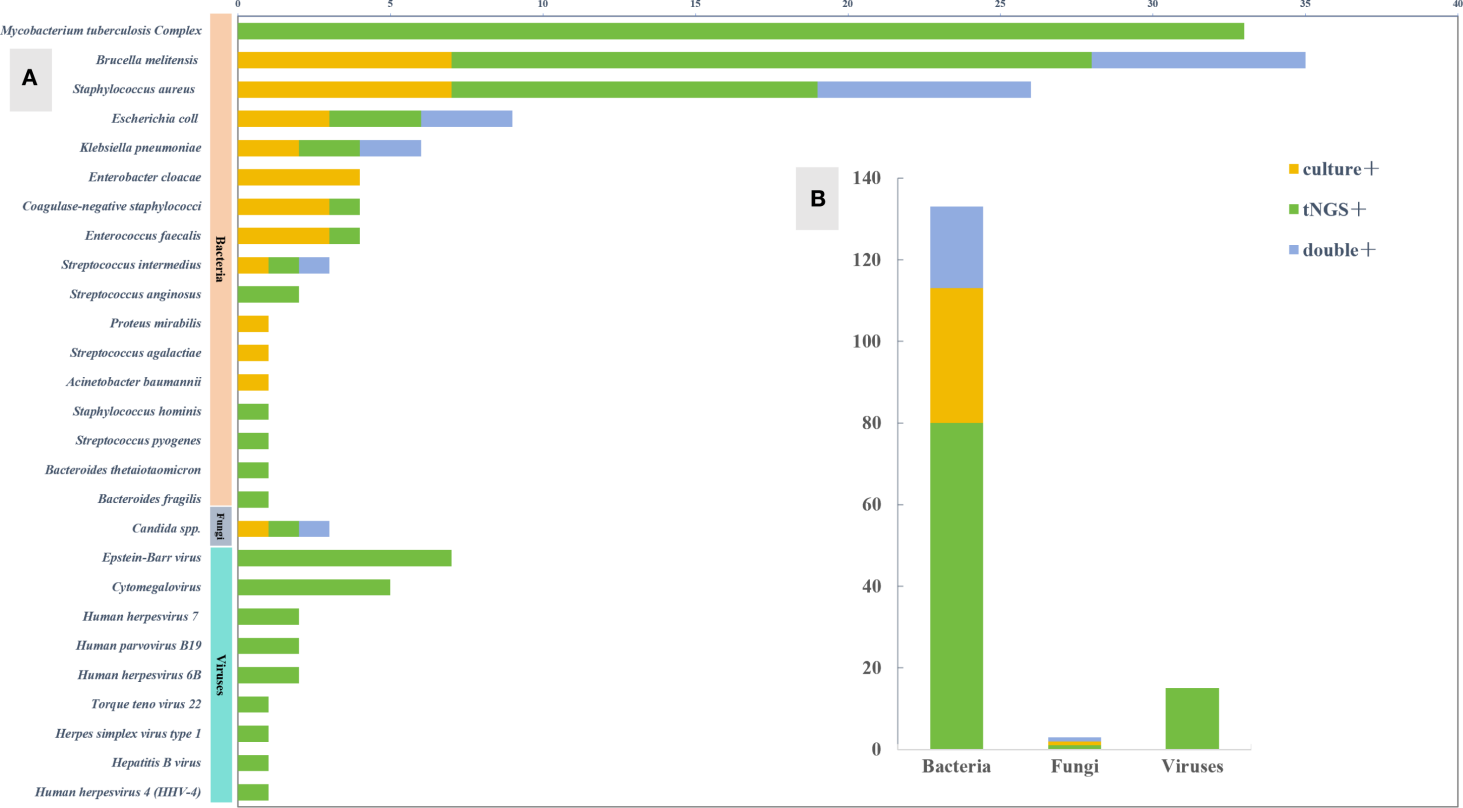
Pathogen spectrum of tNGS and culture. **(A)** Comparison of pathogen spectra detected by tNGS and culture. **(B)** The number of cases of tNGS and culture detected in different types of microorganisms.

Among the 21 samples that were positive for both tNGS and culture, 15 cases (71.4%) were a complete match, 2 cases (9.5%) were a partial match, and 4 cases (19.0%) did not match (**Figure 3**). To provide a clearer overview, the partial and mismatched results between tNGS and culture are summarized in **Supplementary Table 1**. The overall consistency between the two methods, excluding MTBC, was moderate (Cohen’s κ=0.536, *P<*0.001).

Limitations of MTBC Detection: Due to the requirement of BSL-3 laboratory support for Mycobacterium culture, our hospital has limited MTBC culture technology; therefore, Xpert MTB/RIF was used for supplementary detection in clinically suspected tuberculosis patients, confirming 29 cases (82.9%, 29/35), which was highly consistent with tNGS results (κ=0.812, *P<*0.001) (**Table 2**).

**Table 2:** The accuracy of tNGS in patients with POI.

Diagnostic Method	Sensitivity	Specificity	PPV	NPV
Bacterial Culture	27/87(31.0%)	0	27/27(100.0%)	0
Xpert	29/35(82.9%)	50/52(96.2%)	29/31(93.5%)	50/56(89.3%)
SAT	19/21(90.5%)	65/66(98.5%)	19/20(95.0%)	65/67(97.0%)
tNGS	74/87(85.0%)	0	74/74(100.0%)	0
Pathology	24/87(27.6%)	0	24/24(100.0%)	0

### 3.3 Diagnostic efficacy for tuberculous osteoarticular infection

#### 3.3.1 Among the 35 patients with tuberculous osteoarticular infection

Sensitivity: tNGS (91.43%, 32/35) was significantly higher than Xpert (82.86%, 29/35; χ²=6.12, *P=*0.013) and pathological examination (62.9%, 22/35; χ²=9.84, *P=*0.002);

Specificity: Both tNGS and Xpert were 96.2% (50/52), and pathological examination reached 100% (52/52) (p>0.05);

Diagnostic Accuracy: ROC analysis showed that tNGS (AUC = 0.938, 95%CI: 0.891-0.985) was superior to Xpert (AUC = 0.895, 95%CI: 0.827-0.963) and pathological examination (AUC = 0.819, 95%CI: 0.733-0.905) (Delong test, *P<*0.05) (**Figure 2A**);

Consistency Analysis: tNGS was highly consistent with Xpert (Cohen’s κ=0.877, *P<*0.001) and moderately consistent with pathological examination (κ=0.694, *P<*0.001);

Drug Resistance Gene Detection: tNGS detected 6 cases of drug resistance (2 cases of isoniazid, 2 cases of rifampicin, 1 case of ethambutol, 1 case of streptomycin), and 1 case of multidrug resistance (isoniazid + rifampicin + ethambutol + streptomycin + quinolones) (**Table 3**).

**Table 3:** The accuracy of tNGS in patients with TB.

Diagnostic Method	Sensitivity	Specificity	PPV	NPV
Bacterial Culture	0	0	0	0
Xpert	29/35(82.9%)	50/52(96.2%)	29/31(93.5%)	50/56(89.3%)
Pathology	22/35(62.9%)	52/52(100.0%)	22/22(100.0%)	52/65(80.0%)
tNGS	32/35(91.4%)	50/52(96.2%)	32/34(94.1%)	50/53(94.3%)

### 3.4 Diagnostic efficacy for brucellar osteoarticular infection

#### 3.4.1 Among the 21 patients with brucellar infection

Sensitivity: tNGS (100%, 21/21) was significantly higher than SAT (90.5%, 19/21), bacterial culture (33.3%, 7/21), and pathological examination (9.5%, 2/21) (*P<*0.001);

Specificity: tNGS, bacterial culture, and pathological examination were all 100% (66/66), and SAT was 98.5% (65/66) (*P>*0.05);

Diagnostic Accuracy: tNGS (AUC = 1.000) and SAT (AUC = 0.969, 95%CI: 0.934-1.000) were significantly superior to bacterial culture (AUC = 0.667) and pathological examination (AUC = 0.548) (**Figure 1**);

Consistency Analysis: tNGS was almost completely consistent with SAT (κ=0.937, *P<*0.001), moderately consistent with bacterial culture (κ=0.431, *P=*0.002), and had low consistency with pathological examination (κ=0.138, *P=*0.112) (**Table 4**).

**Table 4:** The accuracy of tNGS in patients with BOI.

Diagnostic Method	Sensitivity	Specificity	PPV	NPV
Bacterial Culture	7/21(33.3%)	66/66(100.0%)	7/7(100.0%)	66/80(82.5%)
SAT	19/21(90.5%)	65/66(98.5%)	19/20(95.0%)	65/67(97.0%)
Pathology	2/21(9.5%)	66/66(100.0%)	2/2(100.0%)	66/85(77.6%)
tNGS	21/21(100.0%)	66/66(100.0%)	21/21(100.0%)	66/66(100.0%)

### 3.5 Diagnostic efficacy for pyogenic osteoarticular infection

#### 3.5.1 Among the 31 patients with pyogenic infection

Sensitivity: tNGS (71.0%, 22/31) was higher than bacterial culture (48.4%, 15/31), but the difference was not statistically significant (χ²=2.85, *P=*0.091);

Specificity: tNGS (98.2%, 55/56) and bacterial culture (92.9%, 52/56) did not have a significant difference (χ²=1.07, *P=*0.301);

Diagnostic Accuracy: tNGS (AUC = 0.846, 95%CI: 0.754-0.938) was significantly superior to bacterial culture (AUC = 0.706, 95%CI: 0.583-0.829) (*P=*0.032);

Consistency Analysis: tNGS was highly consistent with bacterial culture (κ=0.624, *P<*0.001) (**Table 5**);

**Table 5:** The accuracy of tNGS in patients with pyogenic osteoarticular infection.

Diagnostic Method	Sensitivity	Specificity	PPV	NPV
Bacterial Culture	15/31(48.4%)	52/56(92.9%)	15/19(78.9%)	52/68(76.5%)
tNGS	22/31(71.0%)	55/56(98.2%)	22/23(95.7%)	55/64(86.0%)

Drug Resistance Gene Detection: tNGS detected 1 case carrying an extended-spectrum β-lactamase gene (blaCTX-M-15 type).

### 3.6 Comprehensive diagnostic efficacy

#### 3.6.1 tNGS demonstrated significant advantages in all three types of osteoarticular infections

**Figure 4:**
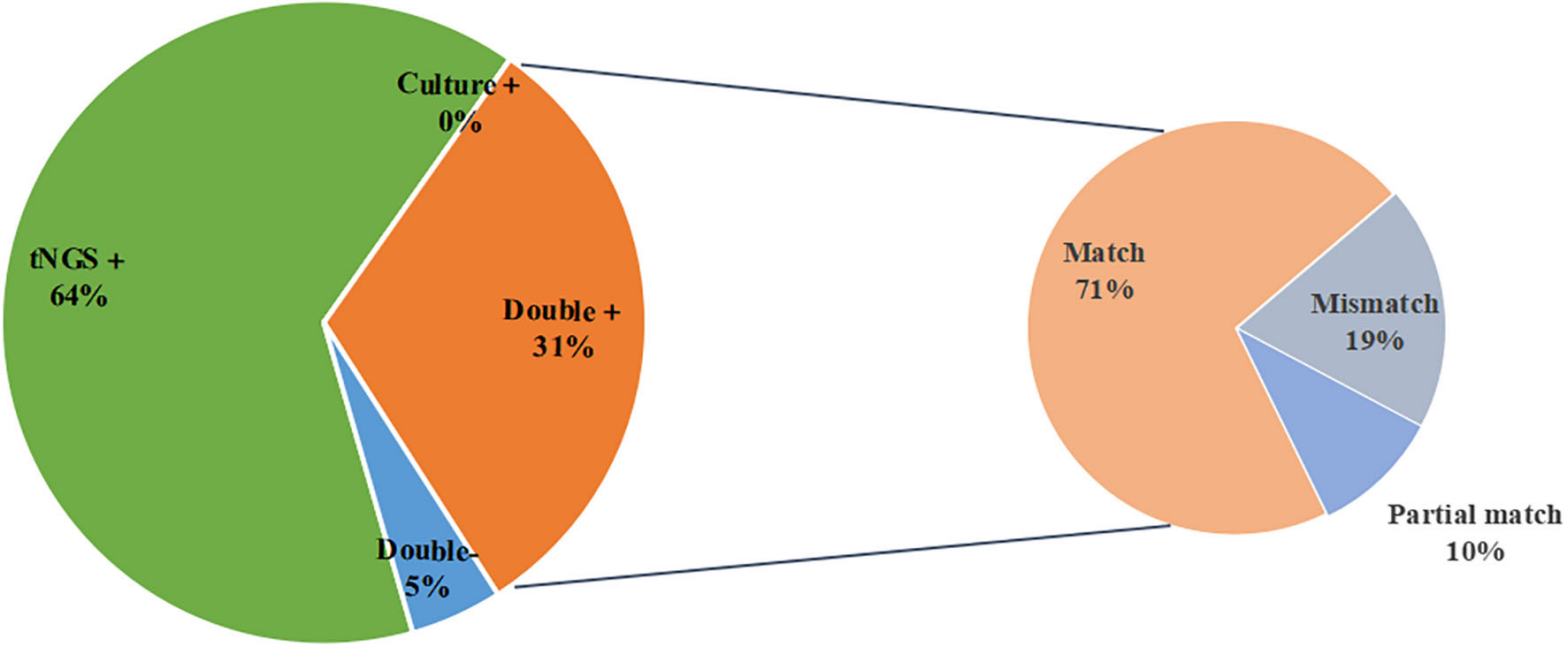
Consistency analysis of tNGS and culture.

Sensitivity Gradient: Brucellar infection (100%) > Tuberculous osteoarticular infection (91.43%) > Pyogenic infection (71.0%);

Diagnostic Accuracy: Brucellar infection (AUC = 1.000) > Tuberculous osteoarticular infection (AUC = 0.938) > Pyogenic infection (AUC = 0.846);

Additional Value: Simultaneously detected 7 drug resistance-related genes (including 6 cases of MTB resistance and 1 case of ESBLs gene), covering first-line anti-tuberculosis drugs and β-lactam antibiotics. In addition, in this study, the average time to obtain culture results was 7.27 days, while the average time to obtain tNGS test results was significantly shorter, at only 2.62 days. These findings are consistent with other relevant studies, indicating that tNGS technology can detect pathogens in POI within a shorter time frame (**Figure 4**).

**Figure 5:**
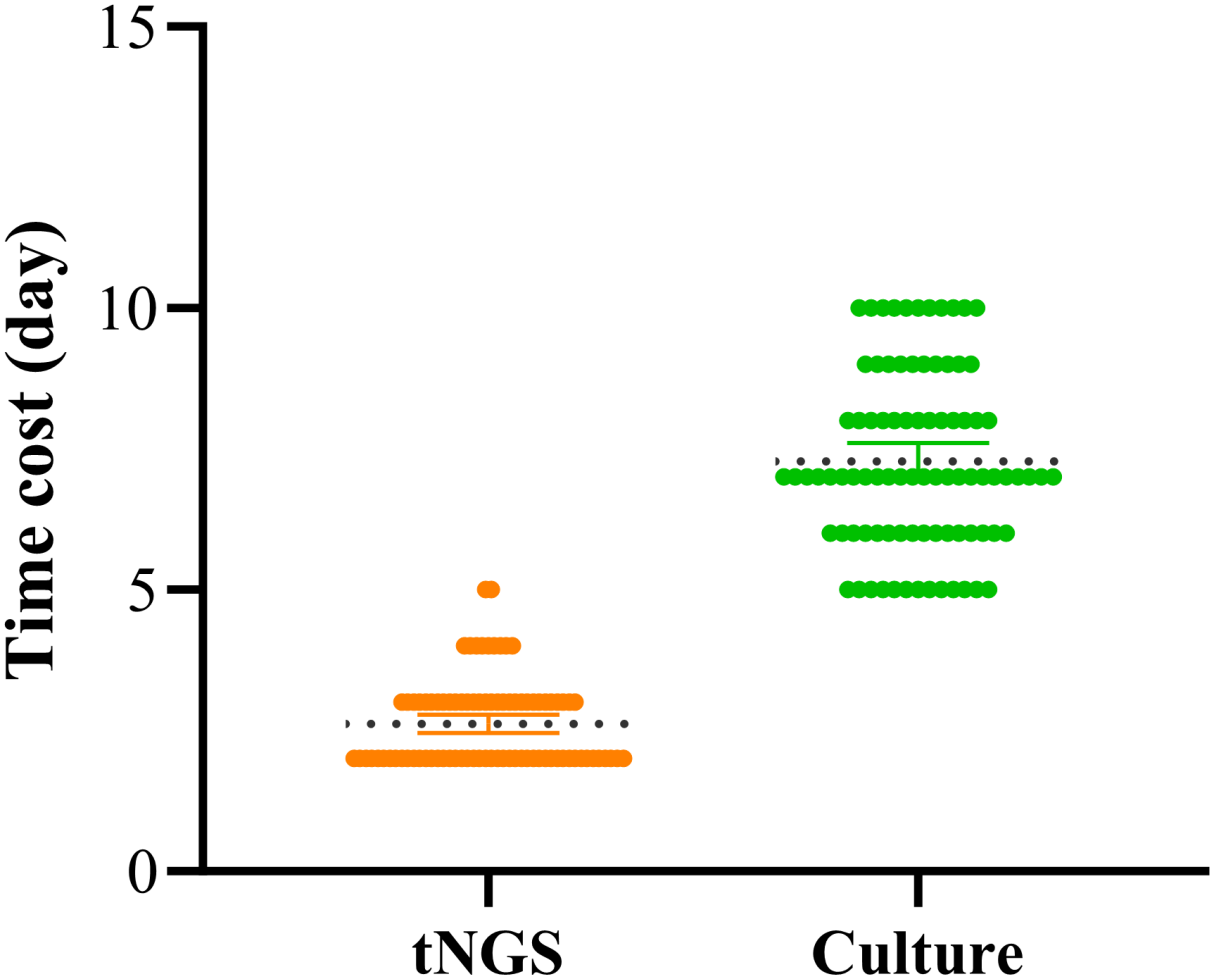
Time cost of tNGS *vs* culture.

Compared with traditional methods (bacterial culture, SAT, and pathological examination), tNGS shows statistically and clinically significant improvements in sensitivity (mean 87.8% *vs*. 42.3%), AUC value (mean 0.928 *vs*. 0.712), and time cost (**Figure 5**).

## 4 Discussion

Bone and joint infections are very destructive and difficult to treat in orthopaedic clinics, there are problems such as specimens are difficult to obtain and patients often receive relevant medications, further reducing the production of bacteria difficult to diagnose accurately. And delayed diagnosis can lead to diagnosis of neurological symptoms and other serious consequences such as paraplegia, amputation, and chronic sensitisation leading to prolonged hospitalisation and even multiple surgeries. For bone and joint infections, an accurate and rapid pathogenetic diagnosis in order to develop an appropriate antibiotic regimen is essential to shorten the duration of the infection and even increase the cure rate. Current diagnosis depends on clinical signs and symptoms, laboratory as well as imaging tests and pathogen detection. Clinically relevant diagnostic techniques alone do not provide a definitive diagnosis, and pathogen testing is indispensable. However, existing pathogen tests, such as bacterial culture, have low detection rates and long detection cycles, which cannot meet the needs of rapid diagnosis of bone and joint infections, which is undoubtedly a great challenge for clinicians. In recent years, the development of second-generation sequencing (NGS) technology has significantly improved the positive detection rate of pathogenic bacteria in bone and joint infections, especially the macro gene second-generation sequencing technology. Relevant literature reports that mNGS can be used to identify spinal infection pathogens and is important in guiding the use of medication (Wang et al., 2023), and that it has a significant advantage over bacterial culture in the diagnostic process in comparison to sensitivity as well as specificity (Ma et al., 2022). However, the current cost of mNGS is much higher than that of bacterial culture and histopathology, which limits its widespread use in clinical practice. In the relevant literature, tNGS has been reported to be valuable for the detection of pathogens as well as drug resistance in related infectious diseases, and the related cost is much lower than that of mNGS (Chen et al., 2024). Therefore, exploring the diagnostic value of tNGS in bone and joint infections is necessary. However, there is little literature on the value of tNGS in POI. In addition to mNGS and tNGS, 16S rDNA broad-range PCR has also been widely applied, particularly in culture-negative infections. This method provides a rapid and sensitive diagnostic approach and is especially useful for detecting slow-growing or difficult-to-culture pathogens that may be missed by conventional techniques (Wang et al., 2020). Recent studies indicate that 16S PCR should not be considered superior to mNGS but rather complementary: it offers a fast and cost-effective bacterial assay, whereas mNGS provides broader pathogen coverage at higher cost. Although 16S PCR was not performed in our study due to its exploratory design and resource limitations, we recognize its potential clinical value and highlight it in the Discussion as an important complementary tool.

Diagnosis as well as treatment of bone and joint tuberculosis infection is also relatively complex, and there are many methods for the diagnosis of *Mycobacterium tuberculosis* pathogens in the clinic (Cabibbe et al., 2020), such as: specific mycobacterial medium culture, X-pert and other protocols, but all of them have more or less defects (Fitzgibbon et al., 2021). Some scholars sequenced 43 sputum specimens for *Mycobacterium tuberculosis* using tNGS technology and concluded that sputum sequencing has the potential to provide a more comprehensive and rapid drug resistance detection than sputum culture (Yang et al., 2018). Relevant literature reported that in paediatric TB patients, tNGS has higher sensitivity but lower specificity than X-pert in diagnosing TB in children. tNGS is more sensitive than X-pert for gastric aspirate and sputum (Zheng et al., 2024). Patients with osteoarticular TB infected with *Mycobacterium tuberculosis* complex need to be treated with antibiotic combinations. However, the usual anti-tuberculosis regimen is not effective against multidrug and extensively drug-resistant MTBC. While drug sensitivity testing for MTBC takes weeks to months to complete, tNGS can rapidly and efficiently detect drug-resistant genes and is a sensitive, reliable, and cost-effective method for detecting drug-resistant TB directly from the original specimen in a clinical or diagnostic laboratory setting, which is a good guide to the treatment of drug-resistant TB (Murphy et al., 2023; Wu et al., 2022). In this study, tNGS was used to detect two cases of isoniazid resistance, two cases of rifampicin resistance, one case of ethambutol resistance, one case of streptomycin resistance, and five cases of pyrazinamide resistance in patients with osteoarticular tuberculosis, and one of these patients had concurrent isoniazid, rifampicin, ethambutol, streptomycin, and quinolone drug resistance. In addition to detecting resistance mutations in Mycobacterium tuberculosis complex, our study also demonstrated that tNGS can reveal resistance determinants in other pathogens relevant to osteoarticular and periprosthetic infections. For example, extended-spectrum β-lactamase (ESBL) genes such as blaCTX-M-15 were identified in Gram-negative isolates. These findings are clinically meaningful, as they allow physicians to refine antibiotic regimens: narrowing broad-spectrum empirical therapy when resistance is absent, or escalating to specific anti-ESBL regimens when resistance genes are present. Thus, tNGS not only facilitates rapid pathogen identification but also contributes to the optimization of antimicrobial strategies across a broader spectrum of infections. Nevertheless, we note that phenotypic susceptibility confirmation was not consistently available in our cohort, and we highlight this as a limitation that should be addressed in future prospective studies.

Brucellosis is a common zoonosis affecting 500,000 people annually. The nervous system is affected in 4-7% of cases. The disease often invades the spine, with a 2-30% incidence involving the skeleton (Laine et al., 2022). Its diagnosis is mainly based on bacterial cultures, but they have a high number of false-negative results. Currently, B. burgdorferi agglutination test on different species of organisms is the main means of diagnosis. It has been reported in the literature that B. burgdorferi agglutination test has high sensitivity as well as specificity for the diagnosis of brucellosis (Baum et al., 1995). Nucleic acid amplification test is a highly sensitive, specific and safe test for rapid diagnosis of the disease. Patients who have been reported to have fully recovered may continue to have positive molecular test results for a long period of time (Elbehiry et al., 2023). Research has demonstrated that next-generation sequencing (NGS) of cerebrospinal fluid (CSF) exhibits strong capability in the diagnosis of neurobrucellosis, enabling rapid and accurate detection of the pathogen (Yu et al., 2023). Whole genome sequencing techniques identified mprF, bepG, bepF, bepC, bepE and bepD in all isolates, but failed to identify other classical resistance genes. For tNGS pathogenetic diagnosis of brucellosis, there is no report in the literature. In contrast, the present study compared tNGS with the B. burgdorferi agglutination test as well as bacterial culture and pathology, and demonstrated that tNGS had a higher diagnostic efficacy in diagnosing B. burgdorferi osteoarticular infections, but there was no significant difference with the B. burgdorferi agglutination test.

Beyond tuberculosis and brucellosis, our study demonstrated that tNGS detected a variety of pyogenic bacteria, including *Staphylococcus aureus*, *Escherichia coli*, *Klebsiella pneumoniae*, and *Streptococcus* spp., while simultaneously identifying resistance genes such as blaCTX-M-15. These findings indicate that tNGS provides valuable guidance for individualized therapy in non-tuberculous osteoarticular and periprosthetic infections. Previous studies have also reported that tNGS can effectively identify pathogens in periprosthetic infections with higher sensitivity and specificity than bacterial culture, and no significant difference with macro gene second-generation sequencing, but compared with mNGS, it has the advantages of low cost and fast turnaround time, and tNGS can provide information on patients’ drug resistance, and comprehensively speaking, tNGS has high diagnostic value (Dadar et al., 2023). In the present study, the effectiveness of tNGS in the diagnosis of non-*Mycobacterium tuberculosis* complex and non-*Brucella* spp. infections was evaluated. bone and joint infections was rarely reported in the literature. tNGS was superior to bacterial culture in terms of diagnostic sensitivity and specificity as well as the AUC value of the ROC curve analysis in the present study, but due to the lack of conditions, the comparative diagnosis of mNGS was not carried out. it is known that, although mNGS has high sensitivity as well as specificity, but there are disadvantages such as high cost, which is burdensome for patients.

Targeted second-generation sequencing technology has not only been remarkably effective in infectious diseases, especially in the identification of pathogenic microorganisms, but also contributes greatly to the diagnosis and treatment of cancer, diagnosis of genetic disorders, breed identification and selection of genomes, etc (Colclough et al., 2022; Kawata et al., 2021; Wang et al., 2021). tNGS may continue to change the existing mode of scientific research and clinical application, and make a significant contribution to the detection of clinical medicine, and the prevention and control of infectious diseases (Yin et al., 2024; Zhang et al., 2024).

This study still has some limitations. Firstly, this study was a single-centre study, and some clinical information before sample collection may not be perfect; secondly, as most patients attending this ward were diagnosed with bone and joint infections, resulting in non-enrolled patients with non-bone and joint infections, which may have biased the results to a certain extent, and affected the precision and reliability of the conclusions. Continued large-sample multicentre studies are needed in the future to improve the reliability of the findings.

This study demonstrates that targeted next-generation sequencing (tNGS) has high sensitivity (71.0%-100%) and specificity (96.2%-100%) in the diagnosis of primary osteoarticular infections (POI), with an AUC value (0.846-1.000) significantly superior to traditional microbiological methods (*P<*0.05). By simultaneously detecting drug-resistant mutations in *Mycobacterium tuberculosis* complex (MTBC) (such as rpoB S450L, katG S315T) and ESBLs resistance genes (blaCTX-M-15), tNGS provides molecular evidence for personalized anti-infective therapy, offering clear clinical translational value.

Limitations of this study include: (1) Sample bias: The single-center retrospective design and limited sample size (n=87) may lead to selection bias and insufficient statistical power (power=0.78); (2) Absence of control group: The lack of a non-infectious bone disease control cohort (such as bone tumors, autoimmune arthritis) may overestimate specificity; (3) Technical validation gap: Due to experimental constraints, no head-to-head comparison with metagenomic next-generation sequencing (mNGS) was conducted.

Future research should involve multicenter prospective studies (target sample size ≥300) and optimize the following aspects: (1) Standardization of testing procedures: Establish SOPs for bone tissue DNA extraction and resistance gene analysis; (2) Cost-effectiveness analysis: Evaluate the health economic value of tNGS in the early diagnosis of POI; (3) Improvement of resistance databases: Integrate WHO catalogs of resistance mutations (such as the MTBPO platform) to enhance the accuracy of resistance prediction.

## 5 Conclusion

This study, through a multi-dimensional comparison of detection methods, has confirmed that tNGS technology, with its high efficiency, broad coverage, and accuracy, can simultaneously achieve the identification of POI pathogens and the detection of resistance genes, demonstrating significant advantages in clinical diagnosis. The technology has important guiding value for optimizing anti-infective treatment regimens and is recommended for widespread adoption as a first-line molecular diagnostic tool for POI.

## Data Availability

The original contributions presented in the study are included in the article/**Supplementary Material**. Further inquiries can be directed to the corresponding authors.

## References

[B1] Babic M. Simpfendorfer C. S. (2017). Infections of the spine. Infect. Dis. Clin. North. Am. 31, 279–297. doi: 10.1016/j.idc.2017.01.003, PMID: 28366222

[B2] Bagcchi S. (2023). Who’s global tuberculosis report 2022. Lancet Microbe 4, e20. doi: 10.1016/S2666-5247(22)00359-7, PMID: 36521512

[B3] Baum M. Zamir O. Bergman-Rios R. Katz E. Beider Z. Cohen A.. (1995). Comparative evaluation of microagglutination test and serum agglutination test as supplementary diagnostic methods for brucellosis. J. Clin. Microbiol. 33, 2166–2170. doi: 10.1128/jcm.33.8.2166-2170.1995, PMID: 7559970 PMC228357

[B4] Cabibbe A. M. Spitaleri A. Battaglia S. Colman R. E. Suresh A. Uplekar S.. (2020). Application of targeted next-generation sequencing assay on a portab le sequencing platform for culture-free detection of drug-resistant tuberculosis from clinical samples. J. Clin. Microbiol. 58, e00632-20. doi: 10.1128/JCM.00632-20, PMID: 32727827 PMC7512157

[B5] Chen Q. Yi J. Liu Y. Yang C. Sun Y. Du J.. (2024). Clinical diagnostic value of targeted next−generation sequencing for infectious diseases (review). Mol. Med. Rep. 30, 153–163. doi: 10.3892/mmr.2024.13277, PMID: 38963022

[B6] Colclough K. Ellard S. Hattersley A. Patel K. (2022). Syndromic monogenic diabetes genes should be tested in patients with a clinical suspicion of maturity-onset diabetes of the young. Diabetes. 71, 530–537. doi: 10.2337/db21-0517, PMID: 34789499 PMC7612420

[B7] Dadar M. Alamian S. Brangsch H. Elbadawy M. Elkharsawi A. R. Neubauer H.. (2023). Determination of virulence-associated genes and antimicrobial resistance profiles in brucella isolates recovered from humans and animals in Iran using ngs technology. Pathogens. 12 (1), 82. doi: 10.3390/pathogens12010082, PMID: 36678430 PMC9865427

[B8] Deng Z. Li C. Wang Y. Wu F. Liang C. Deng W.. (2023). Targeted next-generation sequencing for pulmonary infection diagnosis in patients unsuita ble for bronchoalveolar lavage. Front. Med. 10. doi: 10.3389/fmed.2023.1321515, PMID: 38179267 PMC10764475

[B9] Deurenberg R. H. Bathoorn E. Chlebowicz M. A. Couto N. Ferdous M. Garcia-Cobos S.. (2017). Application of next generation sequencing in clinical microbiology and infection prevention. J. Biotechnol. 243, 16–24. doi: 10.1016/j.jbiotec.2016.12.022, PMID: 28042011

[B10] Dorman S. E. Schumacher S. G. Alland D. Nabeta P. Armstrong D. T. King B.. (2018). Xpert mtb/rif ultra for detection of mycobacterium tuberculosis and rifampicin resistance: a prospective multicentre diagnostic accuracy study. Lancet Infect. Dis. 18, 76–84. doi: 10.1016/S1473-3099(17)30691-6, PMID: 29198911 PMC6168783

[B11] Elbehiry A. Aldubaib M. Marzouk E. Abalkhail A. Almuzaini A. M. Rawway M.. (2023). The development of diagnostic and vaccine strategies for early detection and control of human brucellosis, particularly in endemic areas. Vaccines. 11 (3), 654. doi: 10.3390/vaccines11030654, PMID: 36992237 PMC10054502

[B12] Fitzgibbon M. M. Roycroft E. Sheehan G. Mc L. A. Quintyne K. I. Brabazon E.. (2021). False detection of rifampicin resistance using xpert((r)) mtb/rif ultra assay due to an a451v mutation in mycobacterium tuberculosis. JAC-Antimicrob. Resist. 3, dlab101. doi: 10.1093/jacamr/dlab101, PMID: 34386770 PMC8355037

[B13] Gaston D. C. Miller H. B. Fissel J. A. Jacobs E. Gough E. Wu J.. (2022). Evaluation of metagenomic and targeted next-generation sequencing workflows for detection of respiratory pathogens from bronchoalveolar lavage fluid specimens. J. Clin. Microbiol. 60, e52622. doi: 10.1128/jcm.00526-22, PMID: 35695488 PMC9297812

[B14] Henry M. W. Miller A. O. Walsh T. J. Brause B. D. (2017). Fungal musculoskeletal infections. Infect. Dis. Clin. North. Am. 31, 353–368. doi: 10.1016/j.idc.2017.01.006, PMID: 28483045

[B15] Huang C. Huang Y. Wang Z. Lin Y. Li Y. Chen Y.. (2023). Multiplex pcr-based next generation sequencing as a novel, targeted and accurate molecular approach for periprosthetic joint infection diagnosis. Front. Microbiol. 14. doi: 10.3389/fmicb.2023.1181348, PMID: 37275128 PMC10232910

[B16] Jeon C. Y. Murray M. B. (2008). Diabetes mellitus increases the risk of active tuberculosis: A systematic review of 13 observational studies. PloS Med. 5, e152. doi: 10.1371/journal.pmed.005015, PMID: 18630984 PMC2459204

[B17] Kawata E. Lazo-Langner A. Xenocostas A. Hsia C. C. Howson-Jan K. Deotare U.. (2021). Clinical value of next-generation sequencing compared to cytogenetics in patients with suspected myelodysplastic syndrome. Br. J. Haematol. 192, 729–736. doi: 10.1111/bjh.16891, PMID: 32588428

[B18] Laine C. G. Scott H. M. Arenas-Gamboa A. M. (2022). Human brucellosis: widespread information deficiency hinders an understanding of global disease frequency. PloS Neglect. Trop. Dis. 16, e10404. doi: 10.1371/journal.pntd.0010404, PMID: 35580076 PMC9113565

[B19] Ma C. Wu H. Chen G. Liang C. Wu L. Xiao Y. (2022). The potential of metagenomic next-generation sequencing in diagnosis of spinal infection: a retrospective study. Eur. Spine J. 31, 442–447. doi: 10.1007/s00586-021-07026-5, PMID: 34677679

[B20] Murphy S. G. Smith C. Lapierre P. Shea J. Patel K. Halse T. A.. (2023). Direct detection of drug-resistant mycobacterium tuberculosis using targeted next generation sequencing. Front. Public Health 11. doi: 10.3389/fpubh.2023.1206056, PMID: 37457262 PMC10340549

[B21] Wang C. Liu R. Liu Y. Hou W. Wang X. Miao Y.. (2021). Development and application of the faba_bean_130k targeted next-generation sequencing snp genotyping platform based on transcriptome sequencing. Theor. Appl. Genet. 134, 3195–3207. doi: 10.1007/s00122-021-03885-0, PMID: 34117907

[B22] Wang C.-X. Huang Z. Fang X. Li W. Yang B. Zhang W. (2020). Comparison of. broad-range polymerase chain reaction and metagenomic next-generation sequencing for the diagnosis of prosthetic joint infection. Int. J. Infect. dis.: IJID: Off. publ. Int. Soc Infect. Dis. 95, 8–12. doi: 10.1016/j.ijid.2020.03.055, PMID: 32251799

[B23] Wang G. Long J. Zhuang Y. Leng X. Zhang Y. Liu L.. (2023). Application of metagenomic next-generation sequencing in the detection of pathogens in spinal infections. Spine J. 23, 859–867. doi: 10.1016/j.spinee.2023.02.001, PMID: 36773890

[B24] Wilkes R. P. (2023). Next-generation diagnostics for pathogens. Vet. Clin. North. Am. Food. Anim. Pract. 39, 165–173. doi: 10.1016/j.cvfa.2022.09.003, PMID: 36731996

[B25] Wu S. H. Xiao Y. X. Hsiao H. C. Jou R. (2022). Development and assessment of a novel whole-gene-based targeted next-generation sequencing assay for detecting the susceptibility of mycobacterium tuberculosis to 14 drugs. Microbiol. Spectr. 10, e260522. doi: 10.1128/spectrum.02605-22, PMID: 36255328 PMC9769975

[B26] Xu L. Zhou Z. Wang Y. Song C. Tan H. (2022). Improved accuracy of etiological diagnosis of spinal infection by metagenomic next-generation sequencing. Front. Cell. Infect. Microbiol. 12. doi: 10.3389/fcimb.2022.929701, PMID: 36275025 PMC9585211

[B27] Yang Y. Walls S. D. Gross S. M. Schroth G. P. Jarman R. G. Hang J. (2018). Targeted sequencing of respiratory viruses in clinical specimens for pathogen identification and genome-wide analysis. Methods Mol. Biol. 1838, 125–140. doi: 10.1007/978-1-4939-8682-8_10, PMID: 30128994 PMC7121196

[B28] Yin Y. Zhu P. Guo Y. Li Y. Chen H. Liu J.. (2024). Enhancing lower respiratory tract infection diagnosis: implementation and clinical assessment of multiplex pcr-based and hybrid capture-based targeted next-generation sequencing. EBioMedicine. 107, 105307. doi: 10.1016/j.ebiom.2024.105307, PMID: 39226681 PMC11403251

[B29] Yu L. Zhang Z. Zou Y. Qi X. Zhang Y. Bai K.. (2023). Next-generation sequencing in the diagnosis of neurobrucellosis: a case series of eight consecutive patients. Ann. Clin. Microbiol. Antimicrob. 22, 44. doi: 10.1186/s12941-023-00596-w, PMID: 37268917 PMC10239078

[B30] Zhang P. Liu B. Zhang S. Chang X. Zhang L. Gu D.. (2024). Clinical application of targeted next-generation sequencing in severe pneumonia: a retrospective review. Crit. Care 28, 225. doi: 10.1186/s13054-024-05009-8, PMID: 38978111 PMC11232260

[B31] Zhang H. Wang M. Han X. Wang T. Lei Y. Rao Y.. (2022). The application of targeted nanopore sequencing for the identification of pathogens and resistance genes in lower respiratory tract infections. Front. Microbiol. 13. doi: 10.3389/fmicb.2022.1065159, PMID: 36620015 PMC9822541

[B32] Zheng H. Yang H. Wang Y. Li F. Xiao J. Guo Y.. (2024). Diagnostic value of tngs vs xpert mtb/rif in childhood tb. Heliyon. 10, e23217. doi: 10.1016/j.heliyon.2023.e23217, PMID: 38148816 PMC10750055

