# Supplementary material for: The application value of targeted next-generation sequencing in the diagnosis of primary osteoarticular infections: A single-center study
Source: Front Cell Infect Microbiol. 2025 Sep 18;15:1593228. doi: 10.3389/fcimb.2025.1593228 (PMC12488659; doi:10.3389/fcimb.2025.1593228)
Supplement: Supplementary file 1 [file Table1.docx]

Supplementary Table 1. Summary of partial and mismatched cases between tNGS and culture

| Case ID | Infection type | Culture result | tNGS result | Match status |
| --- | --- | --- | --- | --- |
| P01 | Brucella Osteoarticular infection | *Brucella spp.* | *Brucella spp.* | Match |
| P02 | Pyogenic Osteoarticular infection | *Klebsiella pneumoniae* | *Klebsiella pneumoniae* | Match |
| P03 | Pyogenic Osteoarticular infection | *Staphylococcus aureus* | *Staphylococcus aureus* | Match |
| P04 | Tuberculous Osteoarticular infection | *Proteus mirabilis* | *Mycobacterium tuberculosis Complex* | Mismatch |
| P05 | Pyogenic Osteoarticular infection | *Klebsiella pneumoniae* | *Klebsiella pneumoniae* | Match |
| P06 | Pyogenic Osteoarticular infection | *Staphylococcus aureus* | *Staphylococcus aureus* | Match |
| P07 | Pyogenic Osteoarticular infection | *Brucella spp.* | *Brucella spp.* | Match |
| P08 | Brucella Osteoarticular infection | *Staphylococcus aureus* | *Staphylococcus aureus* | Match |
| P09 | Pyogenic Osteoarticular infection | *Acinetobacter baumannii,*  *Enterococcus faecalis,*  *Enterobacter spp.* | *Mycobacterium tuberculosis Complex* | Mismatch |
| P10 | Pyogenic Osteoarticular infection | *Enterococcus faecalis,*  *Enterobacter spp.* | *Staphylococcus hominis,*  *Brucella spp.* | Mismatch |
| P11 | Brucella Osteoarticular infection | *Staphylococcus aureus* | *Staphylococcus aureus* | Match |
| P12 | Pyogenic Osteoarticular infection | *Staphylococcus aureus* | *Staphylococcus aureus* | Match |
| P13 | Pyogenic Osteoarticular infection | *Escherichia coli,*  *Streptococcus agalactiae* | *Escherichia coli,*  *Staphylococcus aureus* | Partial Match |
| P14 | Pyogenic Osteoarticular infection | *Brucella spp.* | *Brucella spp.* | Match |
| P15 | Brucella Osteoarticular infection | *Staphylococcus aureus* | *Staphylococcus aureus* | Match |
| P16 | Pyogenic Osteoarticular infection | *Brucella spp.* | *Brucella spp.* | Match |
| P17 | Pyogenic Osteoarticular infection | *Streptococcus intermedius* | *Streptococcus intermedius* | Match |
| P18 | Brucella Osteoarticular infection | *Staphylococcus aureus* | *Staphylococcus aureus* | Match |
| P19 | Pyogenic Osteoarticular infection | *Candida spp.*  *Enterococcus spp.*  *Coagulase-negative staphylococci* | *Candida spp.* | Partial Match |
| P20 | Pyogenic Osteoarticular infection | *Coagulase-negative Staphylococci* | *Brucella spp.* | Mismatch |
| P21 | Pyogenic Osteoarticular infection | *Brucella spp.* | *Brucella spp.* | Match |
